# 

**Published:** 1824-05

**Authors:** 


### MONTHLY CATALOGUE OF MEDICAL BOOKS.

*** *It having been hinted to us that gentlemen, sending copies of their Works, prefer having their titles given at length, it is our intention, in future, to comply with this suggestion: but it is to be observed, that no books can be entered on this List except those sent to us for the purpose;—finding, in the lists hitherto transmitted, that the names of works have frequently been given as published, which have not appeared for weeks, or even months after.*

A Treatise on Syphilis; exhibiting the Advantages of large Doses of the Sub-muriate of Mercury in the Cure of that Disease; also an Inquiry into the Modus Operandi of Mercury, illustrated by Experiments. By JAMES BOYLE, Esq. one of the Surgeons to the United Institution of London and Westminster for the Treatment of Lying-in Women and Children; Author of "a Treatise on the Epidemic Cholera of India," and "Letters on the Prevention and Cure of Diseases peculiar to hot and cold Countries, &c.—London, 1824. pp. 166.

A Treatise on the Stricture of the Urethra; designed as a Manual for the Treatment of that Complaint, and addressed chiefly to Students and junior Practitioners. By GEORGE MACILWAIN, Member of the Royal College of Surgeons, Surgeon to the Finsbury Dispensary, Member of the Medico-Chirurgical Society of London, and late Surgeon to the City-of-London Truss Society.—London, 1824. pp. 138.

An Appeal to the Public, and to the Legislature, on the Necessity of affording dead Bodies to the Schools of Anatomy, by Legislative Enactment. By WILLIAM MACKENSIE, Andersonian Professor of Anatomy and Surgery; Member of the Royal College of Surgeons, London, the Faculty of Physicians and Surgeons of Glasgow, and the Medical and Chirurgical Society of London; and Corresponding Member of the Med. and Chir. Society of Edinburgh.—Glasg. 1824. pp. 36.

The Chemical Decompositions of the Pharmacopœia Collegii Regalis Medicorum Londinensis M.DCCCXXIV. To which are added, Tables of the Materia Medica. Intended for the use of Students preparing for their Examination at Apothecaries' Hall. By JONATHAN PEREIRA, Fellow of the Medical, and Member of the Meteorological, Societies of London; Apothecary to the General Dispensary, &c. —London, 1824. pp. 43.

The Apothecary's Chart; showing at one view the Formulæ of all the Preparations in the London Pharmacopœia of 1824, usually made up by the Apothecary or retail Druggist. With Tables of the Weights and Measures; the Quantity of Opium, Mercury, &c. contained in particular Compounds; and a Vocabulary of Words and Contractions used in the Chart. By a Retail Chemist and Druggist.

Recherches Chemiques sur les Corps Gras d'Origine Animale. Par M. E. CHEVREUL.—Paris, 1823. pp. 484.

Recherches sur le Ramolissement de Cerveau: ouvrage dans lequel on s'efforce de distinguer les diverses Affections de ce Viscere par des Signes charactéristiques. Par LEON ROSTAN, Medicin de l'Hospice de la Vieillesse-Femmes (Salpetrière), Professeur de Medecine Clinique. Second Edition.—Paris, 1823. pp. 503.

Observations sur la Nature et le Traitement de l'Hydropisie. Par M. PORTAL, Premier Medecin du Roi, &c. &c. &c. Tome premier et second.—Paris, 1824.

---

Engravings, illustrative of a Work on the Nature and Treatment of the Distortions to which the Spine and the Bones of the Chest are subject. By JOHN SHAW, Surgeon and Lecturer on Anatomy.—London, by Longman and Co. Folio; 13 Plates, containing 34 Figures. pp. 50.

*** To these splendid Engravings we had occasion to allude in our review of Mr. SHAW's work on the Spine. We find, however, that many additional remarks are made in the letter-press which accompanies them. We shall take an early opportunity of laying these before our readers.

System of Anatomical Plates, with descriptive Letter-press. By JOHN LIZARS, F.R.S. Fellow of the Royal College of Surgeons, and Lecturer on Anatomy and Physiology, Edinburgh. Part IV. *Muscles of the Trunk.*

*** These Plates continue to merit the favourable opinion we have expressed of the preceding Parts.

### NOTICE TO CORRESPONDENTS.

*We have to acknowledge the receipt of* Mr. TOM's *letter. The description which he gave of his case of United Twins, in our March Number, is so distinct, that we have not thought it necessary to have an engraving made from the drawing he had the politeness to send.*

Dr. BALFOUR's *Paper, and Letter of the* 16*th April, have been received. We regret that it was not in our power to comply with his request in the present Number.*

*We regret that we cannot insert* Mr. J.'s *Paper: he will find in our* COLLECTANEA *of this month ample information on the subject of his experiments.*

